# Individual Contributions of Amido Acid Residues Tyr122, Ile168, and Asp173 to the Activity and Substrate Specificity of Human DNA Dioxygenase ABH2

**DOI:** 10.3390/cells12141839

**Published:** 2023-07-13

**Authors:** Anastasiia T. Davletgildeeva, Timofey E. Tyugashev, Mingxing Zhao, Nikita A. Kuznetsov, Alexander A. Ishchenko, Murat Saparbaev, Aleksandra A. Kuznetsova

**Affiliations:** 1 Institute of Chemical Biology and Fundamental Medicine, Siberian Branch of Russian Academy of Sciences, 630090 Novosibirsk, Russia; 2 Department of Natural Sciences, Novosibirsk State University, 630090 Novosibirsk, Russia; 3 Groupe Mechanisms of DNA Repair and Carcinogenesis, CNRS UMR9019, Gustave Roussy Cancer Campus, Université Paris-Saclay, CEDEX, F-94805 Villejuif, France

**Keywords:** DNA repair, DNA dioxygenase ABH2, DNA methylation, conformational dynamics, fluorescent spectroscopy, presteady-state kinetics, stopped-flow

## Abstract

Human Fe(II)/α-ketoglutarate-dependent dioxygenase ABH2 plays a crucial role in the direct reversal repair of nonbulky alkyl lesions in DNA nucleobases, e.g., N^1^-methyladenine (m^1^A), N^3^-methylcytosine (m^3^C), and some etheno derivatives. Moreover, ABH2 is capable of a less efficient oxidation of an epigenetic DNA mark called 5-methylcytosine (m^5^C), which typically is a specific target of DNA dioxygenases from the TET family. In this study, to elucidate the mechanism of the substrate specificity of ABH2, we investigated the role of several active-site amino acid residues. Functional mapping of the lesion-binding pocket was performed through the analysis of the functions of Tyr122, Ile168, and Asp173 in the damaged base recognition mechanism. Interactions of wild-type ABH2, or its mutants Y122A, I168A, or D173A, with damaged DNA containing the methylated base m^1^A or m^3^C or the epigenetic marker m^5^C were analyzed by molecular dynamics simulations and kinetic assays. Comparative analysis of the enzymes revealed an effect of the substitutions on DNA binding and on catalytic activity. Obtained data clearly demonstrate the effect of the tested amino acid residues on the catalytic activity of the enzymes rather than the DNA-binding ability. Taken together, these data shed light on the molecular and kinetic consequences of the substitution of active-site residues for the mechanism of the substrate recognition.

## 1. Introduction

Exposure of the cell to alkylating agents originating from external and internal sources constantly damages DNA, thereby leading to the formation of various alkylated lesions that have a cytotoxic and/or mutagenic effect [1,2,3]. Some alkylating agents are also widely used in antitumor therapies and exert their action by generating cytotoxic lesions in tumor cells [4,5]. At the same time, targeted enzymatic DNA methylation plays an important role in the epigenetic regulation of gene expression [6,7]. There is no doubt in the extreme importance of thorough control over the alkylating damage to DNA bases. The participation of DNA methylation in the processes of DNA damage and in the regulation of DNA functions as an information carrier is well characterized; however, demethylation, which is also a very important process in the modulation of the DNA methylation status, remains to be elucidated.

In the past three decades, three main types of demethylation enzymes have been identified in the cells that protect the genome from any alkylated modifications. These types are methyltransferases, repair DNA glycosylases, and repair and epigenetic DNA dioxygenases [1,2,8,9]. Among these types of enzymes, repair DNA dioxygenases of the AlkB family are of particular interest [10,11] because these enzymes carry out a single enzymatic reaction to direct the catalytical repair of various alkyl DNA lesions, such as *N*^1^-methyladenine (m^1^A) and *N*^3^-methylcytosine (m^3^C), through an oxidative mechanism involving the Fe^2+^ ion, α-ketoglutarate (αKG), and oxygen [12,13,14,15,16,17]. After the catalytic oxidation of the methyl group, the decomposition of the hydroxylated intermediate proceeds with the formation of the undamaged base and formaldehyde. Nine orthologs of *E. coli* AlkB dioxygenase have been found in human cells [9]. Nonetheless, only two human enzymes homologous to AlkB have been identified, ABH2 and ABH3, which are capable of the oxidative demethylation of m^1^A and m^3^C [18,19,20]. It is noteworthy that enzymes AlkB and ABH3 have higher activity toward single-stranded DNA (ssDNA) than dsDNA, whereas ABH2 seems to prefer dsDNA substrates [19,21]. It is known that AlkB and ABH3, but not the ABH2 enzyme, can reverse m^1^A and m^3^C lesions in RNA substrates [19,22]. These data apparently indicate that AlkB and ABH3 predominantly repair DNA during replication, when segments of ssDNA are present, whereas the ABH2 enzyme is considered a DNA-repair enzyme acting primarily during the S phase through the interaction with the proliferating cell nuclear antigen (PCNA) [23,24].

To date, it has been thought that dioxygenase ABH2 is only a repair enzyme that protects genomic DNA from nonbulky alkyl lesions, such as m^1^A, m^3^C, 3-methylthymidine (m^3^T), 1,*N*^6^-ethenoadenine (εA), 3,*N*^4^-ethenocytosine (εC), and *N*^2,3^-ethenoguanine [25,26,27]. On the other hand, a recent study additionally revealed an ability of dioxygenase ABH2 to remove the epigenetic mark 5-methylcytosine (m^5^C) from ssDNA and dsDNA in vitro [28]. A major product of the m^5^C oxidation is 5-hydroxymethylcytosine (hm^5^C), but products of its further oxidation to 5-formylcytosine (f^5^C) and 5-carboxylcytosine (ca^5^C) form only in negligible amounts. Given that the demethylation of m^5^C is an important epigenetic process, the above findings suggest that a living organism can employ several redundant or complementary pathways to control an epigenetic modification. The possible biological significance of the discovered activity may be the implementation of a reserve pathway of epigenetic regulation in addition to the main mechanism, which is realized with the participation of TET family dioxygenases [29,30].

**Figure 1:**
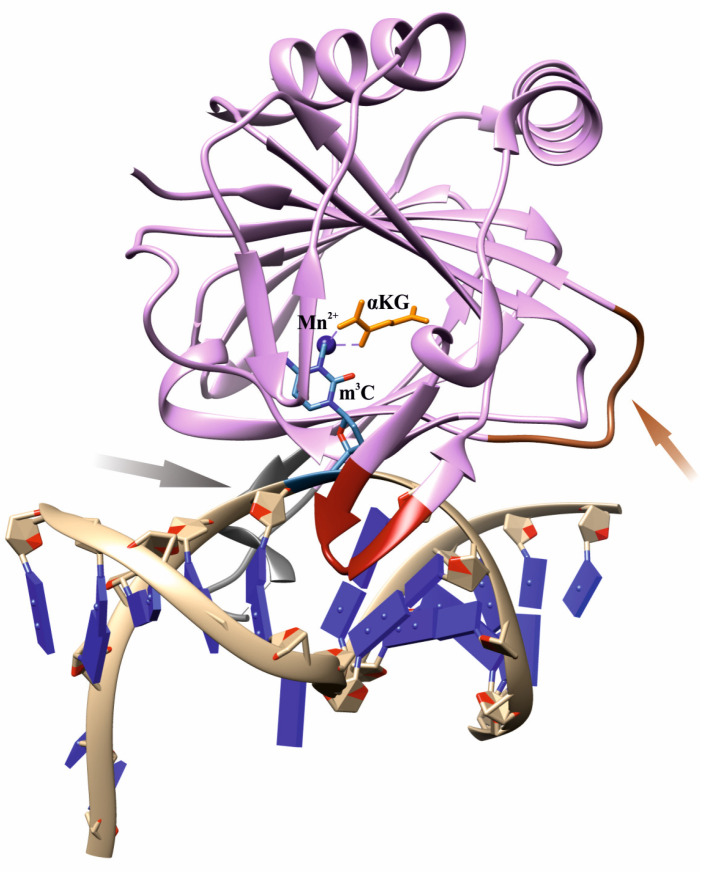
An overview of the structure of a complex of WT ABH2 with DNA containing m^3^C (Protein Data Bank (PDB) ID 3RZJ). Functionally important loops and elements are shown: an intercalating loop containing Val101 and Phe102 (red); a positively charged loop containing Arg241, Lys242, and Lys243 (orange, pointed with an orange arrow); a flexible unresolved loop carrying DNA-binding residues Arg198, Gly204, and Lys205 (gray, pointed with a gray arrow); a metal ion (dark blue); the flipped damaged base inside the active site (blue); αKG (yellow).

Nevertheless, the question of how the ABH2 enzyme discriminates alkylated DNA substrates from other substrates remains a subject of increased interest among researchers. As follows from X-ray data, an effective interaction of AlkB family dioxygenases with DNA and RNA strands is implemented by characteristic loop regions, so-called β-hairpins, whose size and amino acid composition differ substantially among homologs [31,32,33,34,35,36,37]. In particular, ABH2 contains two β-sheet hairpin motifs: the first one intercalates into the DNA duplex by means of residues Val101 and Phe102 and occupies the space left by the damaged base flipped out into the active site, whereas the other motif interacts with the intact DNA strand (Figure 1). To bind the opposite DNA strand, ABH2 uses a short positively charged loop containing Arg241, Lys242, and Lys243, as well as an additional flexible loop carrying the DNA-binding residues Arg198, Gly204, and Lys205. The Fe^2+^ ion is coordinated by the amino acid triad His171, Asp173, and His236.

**Figure 2:**
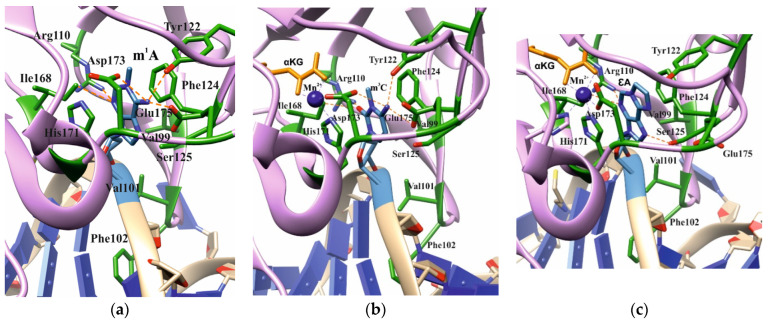
Active-site architecture in the case of m^1^A ((**a**), PDB ID: 3BTY), m^3^C ((**b**), PDB ID: 3RZJ), or εA ((**c**), PDB ID: 3RZK). Amino acid residues coming into contact with the damaged base are shown.

Analysis of the available crystal structures of wild-type (WT) ABH2 with DNA containing m^1^A, m^3^C, or εA inside the active site (Figure 2) allowed us to compile a list of the amino acid residues (Table 1) that form the damaged-base-binding pocket, and thereby could be involved in the damage recognition and control of substrate specificity. It was found that the wall of the damaged-base pocket is formed by Val99, Arg110, Ser125, and Ile168. All types of flipped-out damaged bases are planarly placed between two aromatic residues, Phe124 and His171, resulting in stacking. Additionally, Tyr122, Asp173, and Glu175 come into direct contact with bases m^1^A and m^3^C, but not with εA. Residues Val101 and Phe102 of the DNA-intercalating loop interact with the 5′ and 3′ bases that flank the damaged base.

Several attempts have been made to conduct an extended analysis of the role of the key amino acid residues of the ABH2 enzyme [20,38]. Lee et al. report that substitution D173A inactivates the enzyme toward single-stranded substrates, whereas H236A leads only to some reduction in the activity toward both m^1^A and m^3^C bases. The phenylalanine “finger” Phe102 has been found to be crucial for ssDNA repair; however, the second intercalating residue Val101 shows only reduced demethylating activity toward ssDNA and has no effect on dsDNA. The substitution R110A completely eliminates the enzymatic activity, suggesting that Arg110 is involved in the base-flipping process. It has also been shown that Glu175 and Phe124 contribute to nucleotide-base-specific selection and stabilization in the active site for repair.

To elucidate the mechanism of the substrate specificity of the ABH2 enzyme, analyses of conformational changes occurring in the enzyme–substrate complex during the catalytic cycle have been performed by quantum mechanical/molecular mechanical (QM/MM) modeling [39,40] and by the stopped-flow fluorescence spectroscopy technique [41]. The results indicate that the conformational flexibility of protein domains and of the DNA substrate is important for their effective interaction.

Here, we report the mutational analysis of three of the eleven listed amino acid residues in Table 1, which form contacts with damaged bases inside the active site of ABH2. Tyr122, Ile168, and Asp173 amino acid residues were specifically chosen as the ones able to form direct contacts with the m^1^A or m^3^C lesions from three different spatial points. To elucidate the role of these residues in the mechanism of recognition by the ABH2 enzyme of the damaged methylated base m^1^A or m^3^C or the epigenetic mark m^5^C, we created single-point mutant forms containing the substitution of selected amino acids. In the current study, we decided to adhere to the ‘classic’ switching-off strategy to elucidate the impact of the individual amino acid residues into the catalytic process, thus making the substitution to alanine specifically. Combining computational molecular dynamics (MD) simulations with the experimental detection of DNA binding and enzyme activity allowed us to elucidate the role of the selected amino acid residues in the ABH2 action.

## 2. Results and Discussion

### 2.1. Choosing Potentially Important Amino Acid Residues Forming Specific Contacts with Damaged Bases

The mechanisms of target nucleotide recognition by site-specific enzymes that were elucidated in the last few years for a wide range of enzymes, such as DNA glycosylases [42,43,44,45,46,47] and AP-endonucleases [48,49,50,51,52], suggest that these enzymes utilize a combined approach, including both the indirect recognition of a lesion through the induction of the eversion of the target nucleotide in the active site by DNA bending and the unwinding and subsequent direct verification of the lesion in the active site via the formation of damage-specific contacts. It should be noted that crystal structures enable the analysis of only those contacts between amino acid residues and a damaged base that are already formed inside the binding pocket, whereas the trajectory of the base eversion from the DNA duplex in the active site may also reveal a very important contribution (if any) to the efficiency of damage recognition and substrate specificity.

**Figure 3:**
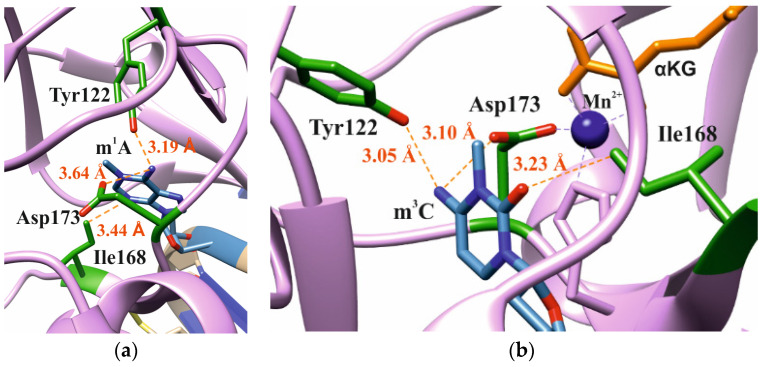
Residues targeted by site-directed mutagenesis in the active site of ABH2 that is in complex with m^1^A-containing DNA ((**a**), PDB ID: 3BTY) or m^3^C-containing DNA ((**b**), PDB ID: 3RZJ).

In our analysis of the structures of the ABH2 enzyme in complex with DNA containing m^3^C (PDB ID: 3RZJ) or m^1^A (PDB ID: 3BTY) as damage, a number of amino acid residues were selected that were of potential interest for studying their role in the step-by-step damaged-base-recognition process. This analysis of the structures of the ABH2–DNA complexes (Figure 3) revealed that amino acid residues Tyr122, Ile168, and Asp173 engage in contacts with both m^3^C and m^1^A (damaged bases) placed inside the active site; these observations supported the possible importance of these residues for the damage-checking mechanism of substrate specificity. Therefore, these amino acid residues were chosen to verify their participation in DNA binding, catalytic complex formation, and catalysis.

### 2.2. MD Simulations of Mutant Forms of the Enzyme

**Figure 4:**
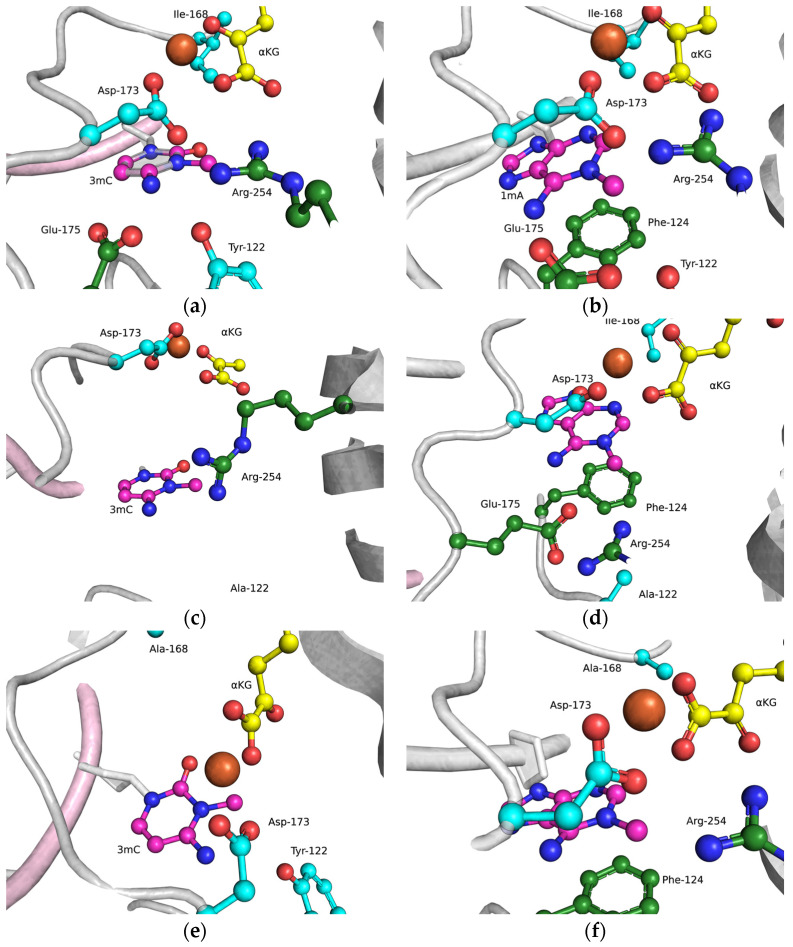

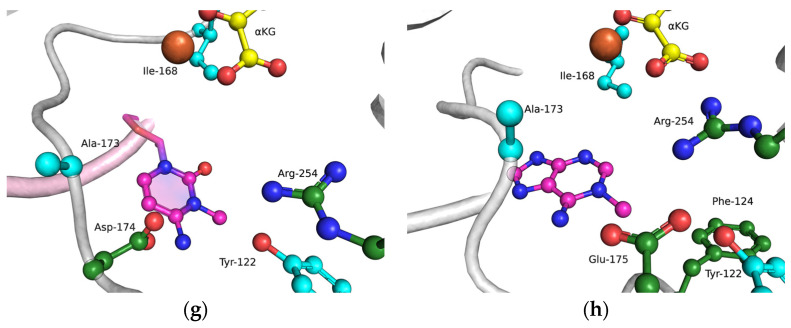
Close-up view of m^3^C (**a**,**c**,**e**,**g**) and m^1^A (**b**,**d**,**f**,**h**) damage-recognition workspace in the active site of WT ABH2 (**a**,**b**) or its mutant forms Y122A (**c**,**d**), I168A (**e**,**f**), or D173A (**g**,**h**), as determined by MD simulations.

The functions of the amino acid residues Tyr122, Ile168, and Asp173 in damage recognition were estimated by MD simulations. ABH2 cross-linked with DNA in the presence of the cofactor αKG and the Mn^2+^ ion (PDB ID: 3RZJ for m^3^C-containing DNA and PDB ID: 3BUC for m^1^A-containing DNA) were used as initial simulation structures [32,39]. The conformational equilibration of these initial structures helped to obtain models of WT ABH2 with DNA containing m^3^C or m^1^A (Figure 4a,b). It turned out that MD relaxation leads to the slight movement of damaged nucleotides within the active site in comparison with crystal structures. Such movement proceeds owing to the formation of set contacts between the damaged-base and active-site amino acid residues, thereby resulting in the optimized positioning of the damaged nucleotides. Then, complexes of ABH2 mutant forms Y122A (Figure 4c,d), I168A (Figure 4e,f), or D173A (Figure 4g,h) with DNA containing m^1^A or m^3^C were modeled to analyze conformational rearrangements induced by the selected amino acid substitution.

The obtained MD model of Y122A (Figure 4c,d) revealed that the substitution of Tyr122 causes a loss of the hydrogen bond with Arg254 and the disappearance of the salt bridge Arg254–Asp173, which is important for the coordination of the Fe^2+^ ion in the active site by Asp173. Such destabilization of the Fe^2+^ ion’s position disturbs the metal ion coordination sphere and most likely should affect the catalytic efficacy.

The substitution of I168A (Figure 4e,f) leads to the widening of the active-site pocket, thereby allowing an αKG molecule to move, rotate, and interact with the Fe^2+^ ion only via the carboxyl group. It should be pointed out that such orientations also occurred in our simulation trajectory for the WT enzyme, albeit for a much shorter period; therefore, the catalytic activity of ABH2 I168A most likely is low, owing to a catalytically “wrong” position of αKG.

In the case of D173A (Figure 4g,h), the stacking of the damaged bases with the Phe124 residue is disturbed owing to a different position of the target nucleotide. Moreover, the undereversion of both tested damaged bases leads to its greater mobility in the active-site pocket. Although the DNA conformation is imperfect for the catalytical state, the Fe^2+^ ion retains its catalytically competent position due to an additional water molecule in place of Asp173.

**Figure 5:**
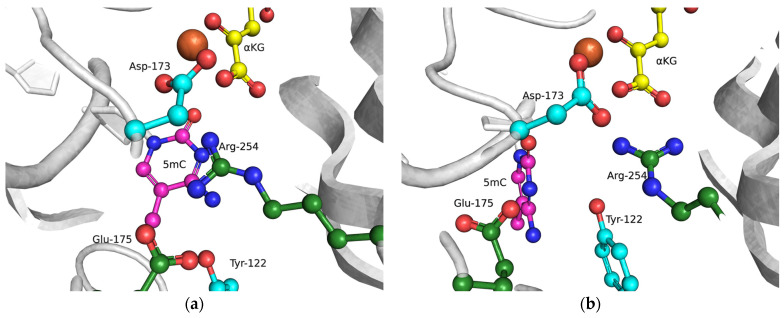
Close-up view of possible positions (“noncatalytic” (**a**) and “catalytic” (**b**)) of an m^5^C base in the active site of WT ABH2.

To clarify the specific features of the substrate-specificity mechanism that allows for the recognition of the epigenetic mark m^5^C in the active site of ABH2, as reported recently [28], MD models of WT ABH2 with DNA containing an m^5^C base were set up (Figure 5). These models enabled us to determine the importance of the contacts of the damaged base with active-site amino acids. It should be noted that the m^5^C base could be positioned in the active site in two alternative ways. In the first (“noncatalytic”) position, the m^5^C base is positioned similarly to the m^3^C base, and thus the methyl group is located far away from the catalytic amino acid triad in a position unsuitable for oxidation. In such an orientation, most of the stabilizing contacts with the damaged base are formed, which retain the base inside the active site. In the other (“catalytic”) position, the *N*-glycosidic bond of m^5^C is rotated to place the methyl group close to the catalytic amino acids. Nonetheless, in this position, there is a loss of many contacts that hold the everted base inside the active site. Taken together, these data suggest that an equilibrium between these positions will significantly affect the ability of ABH2 to catalyze the oxidation of the m^5^C base. The disturbance of the active-site architecture by the selected amino acid substitution could influence this equilibrium, owing to a loss of some specific contacts inside the active site.

### 2.3. Circular Dichroism (CD) Spectra

**Figure 6:**
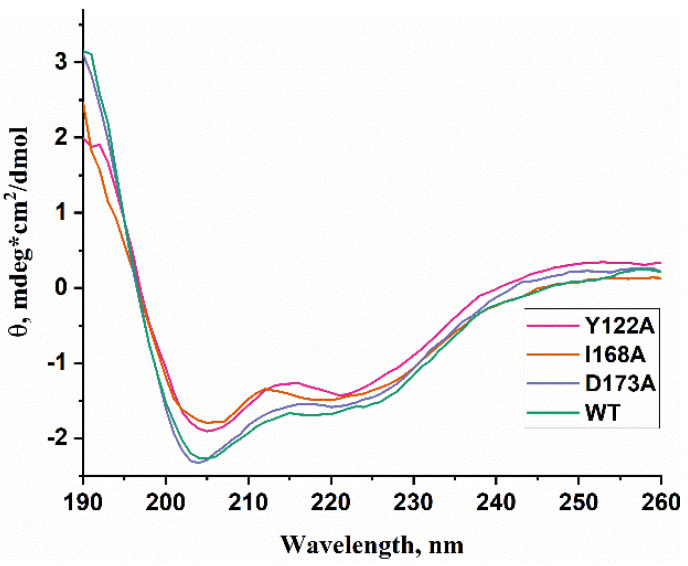
CD spectra of WT ABH2 (green) and of mutant forms featuring the Y122A (pink), I168A (orange), or D173A (purple) substitutions.

WT ABH2 and its mutants featuring the Y122A, I168A, or D173A substitutions were purified to experimentally evaluate the activity of these enzymes toward DNA containing m^3^C, m^1^A, or m^5^C. First of all, CD spectra of these recombinant proteins were recorded to compare the effects of the chosen substitutions on the structure of the enzyme (Figure 6). The results showed similar structural patterns and allowed us to conclude that the selected substitutions do not cause a global alteration of the secondary structure of the protein.

### 2.4. Equilibrium Binding of WT ABH2 or Its Mutant Forms to Methylated DNA

**Figure 7:**
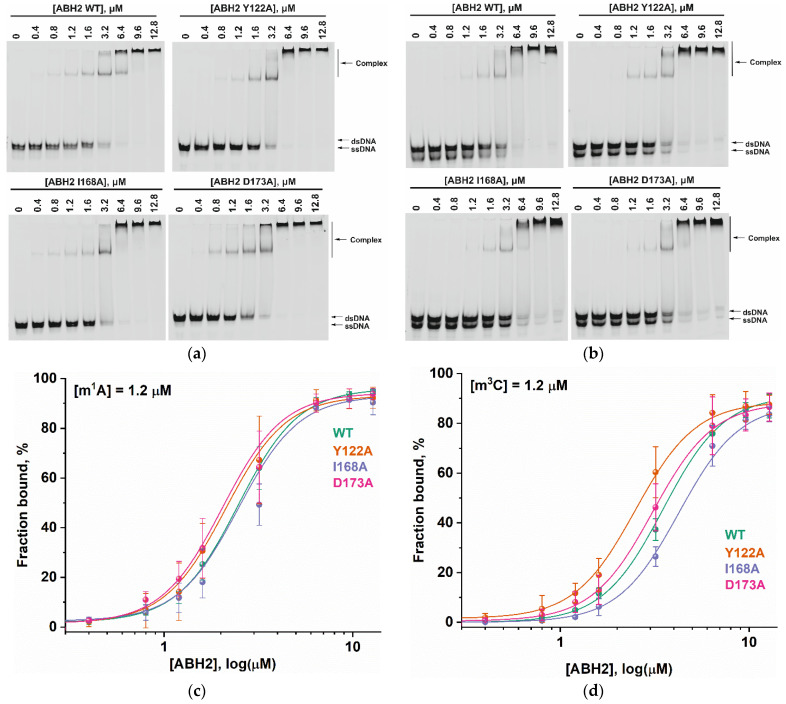
The EMSA of the WT ABH2 enzyme and its mutant forms Y122A, I168A, and D173A. (**a**) EMSA of WT ABH2 or of Y122A, I168A, or D173A with 1.2 µM FAM-labeled m^1^A-containing DNA. (**b**) EMSA of WT ABH2 or Y122A, I168A, or D173A with 1.2 µM FAM-labeled m^3^C-containing DNA. (**c**) Fractions of bound FAM-labeled m^1^A-containing DNA plotted against ABH2 concentrations, with the curve fitting of the obtained data. (**d**) The fractions of bound FAM-labeled m^3^C-containing DNA plotted against ABH2 concentrations, with the curve fitting of the obtained data. In each panel, the concentration of DNA is 1.2 µM, and concentrations of the enzyme are 0, 0.4, 0.8, 1.2, 1.6, 3.2, 6.4, 9.6, and 12.8 µM.

**Figure 8:**
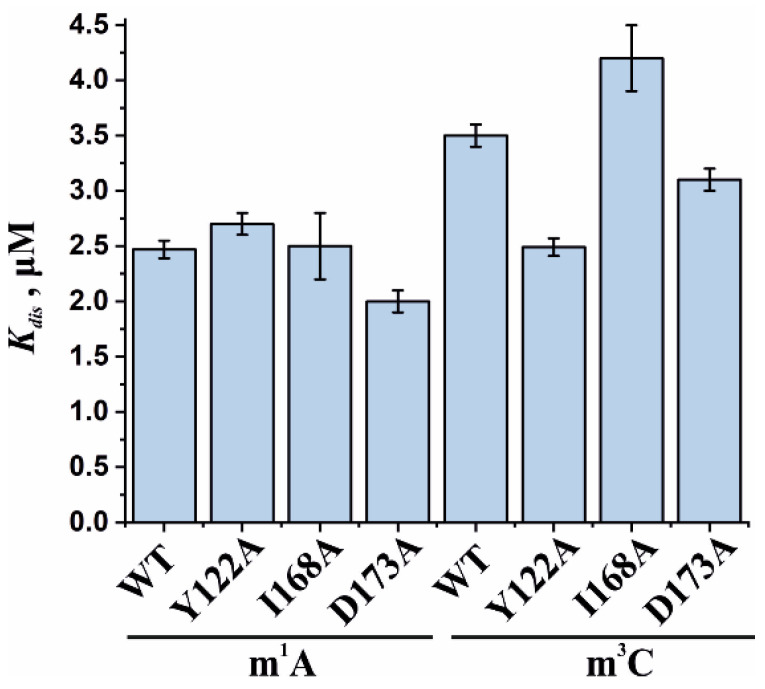
Analysis of the binding affinity of WT ABH2 or its mutant forms Y122A, I168A, and D173A in an EMSA. Dissociation constants (*K_d_*) were determined by the fitting of the experimental data points to the Hill equation (Equation (1)). Error bars represent standard deviation of three technical replicates.

To assess the influence of the amino acid substitutions Y122A, I168A, and D173A on the ability of the enzyme to bind methylated DNA substrates, electrophoretic mobility shift assays (EMSAs) were performed in the presence of 5′-FAM-labeled oligonucleotides containing m^1^A or m^3^C as a lesion (Figure 7a,b). The enzymes were titrated with 17-nt dsDNA containing a methylated nucleotide. Equilibrium dissociation constants (*K_d_*) for complexes of the enzyme with target dsDNA (Figure 7c,d) were calculated by means of Equation (1). Dissociation constants were in a narrow range of 2.1–2.7 µM for the binding to the m^1^A-containing DNA and 2.5–4.2 µM for the binding to the m^3^C-containing DNA (Figure 8). It should be mentioned that these values are in good agreement with data obtained previously in an equilibrium fluorescent titration assay [41].

The obtained *K_d_* values indicate that the WT ABH2 enzyme—just as with its mutant forms—binds to dsDNA containing the methylated base m^1^A with an affinity similar to that for m^3^C-containing dsDNA, with a slight preference for m^1^A. It must be noted that, in the range of *K_d_* values for m^1^A binding (D173A ≤ Y122A < WT ≤ I168A), the greatest value was observed in the case of I168A, and this value was only 1.3-fold greater than the smallest one (in case of D173A), meaning that all of the introduced amino acid substitutions have only a minor influence on the processes involving the binding of an m^1^A-containing substrate. For m^3^C, the impact of the substitution manifested itself as a 1.6-fold difference between the greatest value (for I168A) and the lowest one (for Y122A). It is also worth noting that in both cases—m^1^A- and m^3^C-containing DNA—the substitution of Ile168 weakens the binding affinity of the ABH2 enzyme.

### 2.5. Activity of WT ABH2 and Its Mutant Forms toward DNA Containing m^1^A or m^3^C

**Figure 9:**
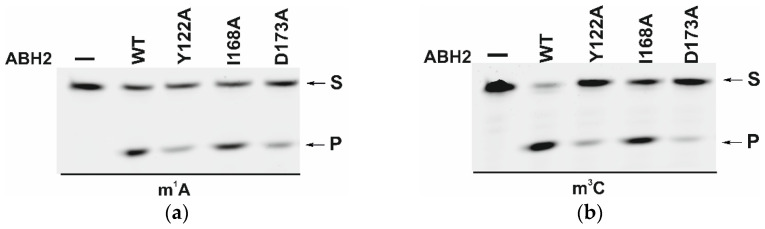

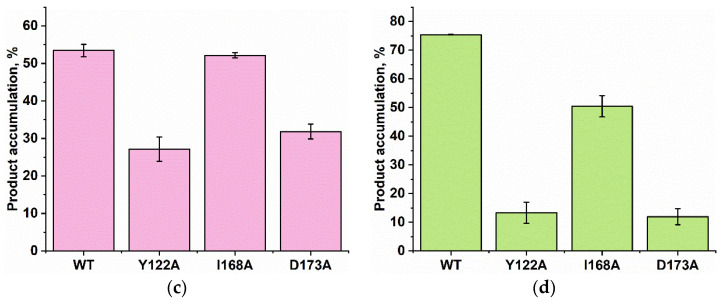
PAGE analysis of the DNA demethylation by ABH2 enzymes during an interaction with the dsDNA substrate containing m^1^A (**a**) or m^3^C (**b**). A comparison of efficacy of demethylation of damaged DNA substrates by ABH2 enzymes during interaction with m^1^A-containing (**c**) or m^3^C-containing DNA (**d**). [Enzyme] = [DNA] = 1.0 µM. T = 37 °C, reaction time = 30 min. S is a substrate, P is a product of the cleavage of the demethylated DNA chain by the restriction enzyme. Levels of accumulation of the product are presented as the average of three experimental values ± SD.

The efficiency of active demethylation of damaged dsDNA substrates by WT ABH2 or its mutant forms Y122A, I168A, or D173A was measured by a restriction-coupled assay followed by gel electrophoresis (Figure 9a,b). The reaction mixture contained equimolar amounts of the enzyme and of its substrate, which was preincubated with Fe^2+^ ions in the presence of αKG. After 30 min, the reaction was terminated, and the products were digested with a specific restriction endonuclease to cleave the product of the active demethylation. The shortened reaction product was then visualized in a gel, and thus the activity of each enzyme on both substrates, m^1^A and m^3^C DNA, was determined.

As shown in Figure 9c,d, WT ABH2 and all mutant forms were able to cleave the dsDNA substrate containing m^1^A or m^3^C. The highest level of product accumulation was observed during the active demethylation of the m^1^A- or m^3^C-containing dsDNA substrate by WT ABH2 (54.0% and 75.4%, respectively; Figure 9c,d). The incomplete substrate conversion could be associated with some degradation of the enzyme, substrate, or one of the cofactors during the long reaction process. Indeed, during the reaction, the concentration of Fe^2+^ ions or O_2_ can substantially decrease in the solution. Additionally, this effect could come out of the inhibition of the enzyme by the products of the reaction, such as succinate. However, this enzyme assay allows us to compare the activity of mutant forms. As such, the substitution of Ile168 has different consequences for the tested lesions: the level of the product accumulation was very similar to that of the WT enzyme for m^1^A (52.1% and 54.0%, respectively), whereas it was low in the case of m^3^C (50% and 75.4%, respectively). As determined by MD simulations (Figure 4), the I168A substitution causes the widening of the active-site pocket and increases the movements of the αKG molecule within the active site. It can be hypothesized that, in the case of the smaller m^3^C base, such increased mobility of αKG leads to the shorter average time of the catalytically optimal orientation of the substrate and cofactor. In the cases of the substitutions Y122A and D173A, the product accumulation for both m^1^A and m^3^C was significantly lower in comparison with the WT enzyme. Nonetheless, the decrease of activity toward m^3^C was again much more drastic, supporting the notion that a disturbance of the Fe^2+^ ion coordination environment plays a more important role for this damaged base. Accordingly, it can be concluded that the substitutions of Tyr122, Ile168, and Asp173 all weaken the preference for m^3^C over m^1^A, seen in WT ABH2.

**Figure 10:**
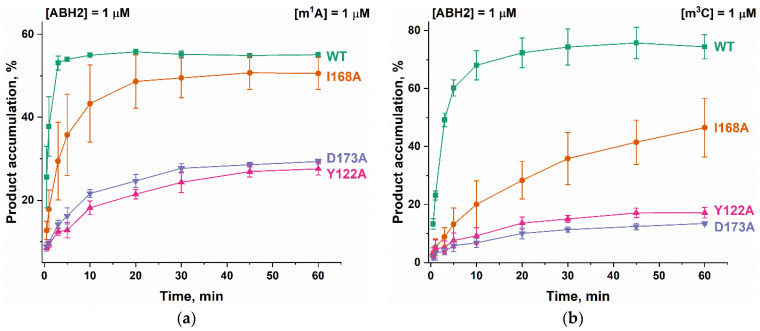
Active demethylation of m^1^A-containing (**a**) or m^3^C-containing (**b**) DNA substrates by WT ABH2, Y122A, I168A, or D173A. [Enzyme] = [DNA] = 1.0 µM. T = 37 °C. Levels of accumulation of the product at each time point are presented as the average of three experimental values ± SD.

To clarify the details of the impact of the amino acid substitutions on specific features of the reaction of the ABH2 enzyme with the methylated DNA substrates, the kinetics of product accumulation during the active demethylation of DNA substrates containing m^1^A or m^3^C by WT ABH2 or its mutant forms were investigated next. Time courses of the substrate demethylation are presented in Figure 10. A comparison of the time courses of the active demethylation of the m^1^A lesion between WT ABH2 and its mutant forms suggested that, despite similar levels of overall product accumulation, the repair of this lesion by the ABH2 I168A mutant form was slower than that by WT ABH2 (Figure 10a). The substitution of Tyr122 or Asp173 leads to the substantial deceleration of the enzyme, thus reducing the overall efficiency, and the effects are quite similar between the two substitutions, indicating that the effects on the catalytic activity of the ABH2 enzyme are nearly identical when these two amino acids are compared. Moreover, the much lower activity of Y122A and D173A mutant forms toward the m^3^C lesion was similar to that for the m^1^A lesion (Figure 10b). The decrease in the initial velocity and overall efficiency of the m^3^C demethylation by the I168A mutant form was well pronounced but not as dramatic as that seen with substitutions Tyr122 or Asp173.

It is interesting to note that the final amount of the overall product accumulation for the WT ABH2 interaction with m^3^C-containing DNA was higher than that for m^1^A-containing DNA, but the rate of the accumulation was slower. The introduction of one of three amino acid substitutions caused a greater decrease of enzymatic activity toward lesion m^3^C than toward m^1^A.

**Figure 11:**
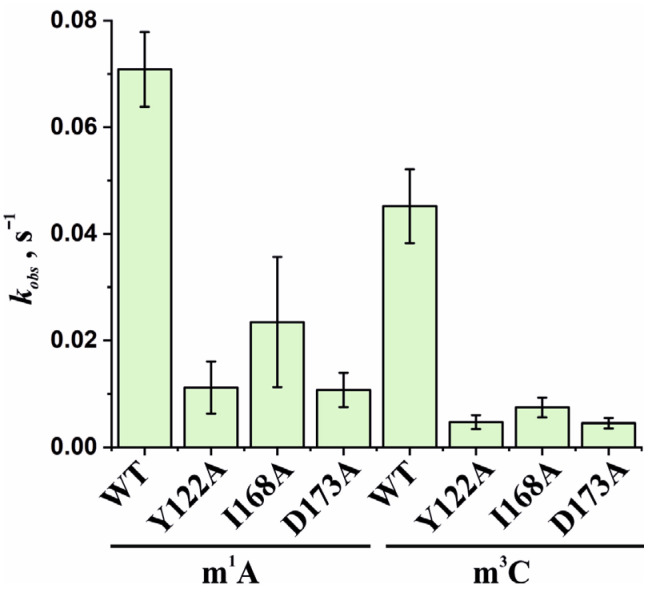
Observed rate constants *k_obs_* characterizing the catalytic activity of WT ABH2 and its mutant forms Y122A, I168A, and D173A toward m^1^A- and m^3^C-containing DNA substrates. The *k_obs_* values were determined by means of Equation (2). Error bars represent the SD determined by mathematical data processing.

Based on the obtained time courses and the EMSA data, the observed rate constants *k_obs_* were determined with the help of Equation (2). The *k_obs_* values characterizing the catalytic repair of the m^1^A- and m^3^C-containing DNA substrates by WT ABH2 or its mutant forms Y122A, I168A, or D173A are presented as a diagram in Figure 11 and are listed in Table 2. Kinetic analysis revealed that, for the ABH2 I168A mutant form, the *k_obs_* values characterizing its catalytic activity toward m^1^A- and m^3^C-containing DNA substrates were 4- and 6-fold lower, respectively, as compared to the WT enzyme (Table 2). The *k_obs_* values of the m^1^A demethylation reactions depended on the enzyme and were in the range between 0.010 s^−1^ for ABH2 D173A and 0.071 s^−1^ for WT ABH2. As expected, the *k_obs_* values for D173A and Y122A (0.013 s^−1^) variants were very close. Just as in the case of m^3^C, the *k_obs_* values for D173A and Y122A were the lowest in the range, and their difference was within the margin of error (0.0045 ± 0.0009 and 0.005 ± 0.001 s^−1^, respectively).

## 3. Conclusions

Mutational analysis of Tyr122, Ile168, and Asp173 residues, which form contacts with damaged bases inside the active site of ABH2, was performed by combining of computational and experimental approaches. These amino acid residues form direct contacts with the m^1^A or m^3^C lesions and, thereby, allow us to estimate their possible role in the recognition of the epigenetic mark m^5^C. Molecular dynamics simulations revealed the impact of these amino acid residues in the catalytic process. In the case of Y122A, a loss of the hydrogen bond between Tyr122 and Arg254 was found, resulting in the disappearance of the salt bridge Arg254–Asp173, which is important for the coordination of the Fe^2+^ ion in the active site by Asp173. The substitution of I168A allowed an αKG molecule to move inside the active site, thereby providing a greater variety of αKG positions. D173A substitutions lead to an increase of the mobility of damaged bases in the active-site pocket, owing to the disturbance of the stacking of m^1^A or m^3^C with the Phe124 residue. The MD models of WT ABH2 with DNA containing an m^5^C base revealed that the m^5^C base could be positioned in the active site in alternative “noncatalytic” and “catalytic” ways. However, in the “catalytic” position, when the methyl group of m^5^C is close to the catalytic amino acids, there is a loss of many contacts that hold the everted base inside the active site.

Experimental EMSA analysis of the binding of WT ABH2 or its mutant forms to methylated DNA revealed only a minor influence of all the tested substitutions on the DNA-binding ability. Indeed, the obtained *K_d_* values were varied between the enzymes within 1.3-fold and 1.6-fold for the DNA containing m^1^A or m^3^C, respectively. However, the analysis of the product accumulation data revealed that, in the case of mutant forms, the rate constant of the product accumulation *k_obs_* was significantly lower as compared to the WT enzyme. Therefore, the obtained data clearly demonstrate the effect of the tested amino acid residues on the catalytic activity of the enzymes rather than the DNA-binding ability. Taken together, these data shed light on the molecular and kinetic consequences of the substitution of active-site residues for the mechanism of the substrate recognition.

## 4. Materials and Methods

### 4.1. Oligodeoxyribonucleotides

The synthesis of the oligodeoxyribonucleotides (Table 3) was performed on an ASM-800DNA/RNA synthesizer (Biosset, Novosibirsk, Russia) by means of standard commercial phosphoramidites and CPG solid supports from Glen Research (Sterling, VA, USA). The oligonucleotides were deprotected according to the manufacturer’s protocols and were purified by high-performance liquid chromatography. Oligonucleotide homogeneity was checked by denaturing 20% PAGE. Concentrations of oligonucleotides were calculated from their absorbance at 260 nm (A_260_). Oligonucleotide duplexes were prepared via the annealing of oligonucleotide strands at a 1:1 molar ratio.

### 4.2. Site-Directed Mutagenesis

This procedure was used to mutate the *ABH2* gene at sites of interest. Primers were synthetized as described above. Three mutant forms were designed by (1) the substitution of a tyrosine residue with alanine at position 122, (2) the substitution of an isoleucine residue with alanine at position 168, or (3) the substitution of an aspartate residue with alanine at position 173. Sequences of the forward primers in question were as follows (reverse primers were complementary to forward primers): 5′-GCTGGGCTGACCGCAACATTTTCAGG-3′ (Y122A), 5′-GATGGCTGTGACCACGCCGGGGAGCAC-3′ (I168A), and 5′-CGGGGAGCACCGAGCAGATGAAAGAGAACTG-3′ (D173A). After PCR, the methylated DNA was removed by MalI (SibEnzyme, Novosibirsk, Russia) digestion. These mixtures were transfected into the *Escherichia coli* strain ElectroMAX™ DH10B (Invitrogen, Waltham, MA, USA). Plasmid DNA was isolated from the resulting cell culture according to the protocol of the diaGene Isolation Kit (Novosibirsk, Russia). The concentration of the isolated plasmid DNA was measured on a NanoPhotometer (Implen, Munich, Germany), and its length was determined in an agarose gel. Sequences of the transformed clones were confirmed by direct sequencing.

### 4.3. Enzyme Purification

**Figure 12:**
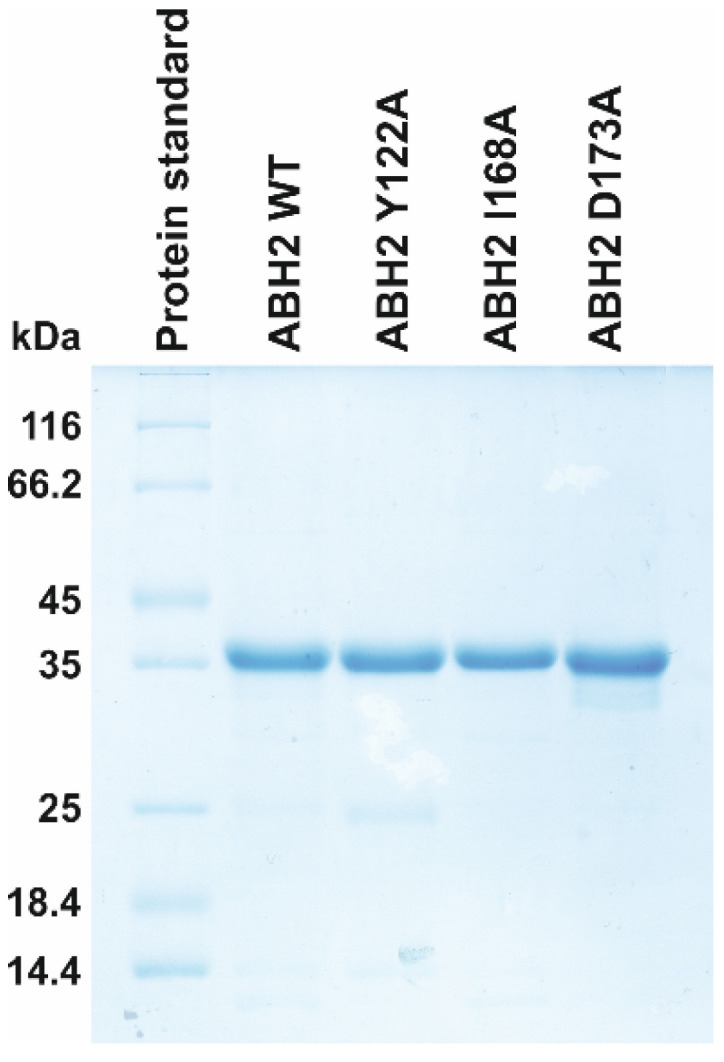
Analysis of protein purity of the WT, Y122A, I168A, and D173A ABH2 enzymes by SDS-PAGE. The proteins were stained with Coomassie Blue R-250 (Sangon Biotech, Shanghai, China).

The WT ABH2 enzyme (ABH2_wt) and its mutant forms featuring substitutions Y122A, I168A, or D173A were isolated from *E. coli* ArcticExpress (DE3) cells (Invitrogen, Waltham, MA, USA) transformed with plasmid pET28c encoding an N-terminal His_6_ tag and the relevant gene. To purify the enzymes expressed as recombinant proteins, 1 L of culture (in YT broth) of *E. coli* cells carrying the desired vector construct was grown with 50 µg/mL kanamycin, 20 µg/mL gentamycin, and 10 µg/mL tetracycline at 37 °C until A_600_ reached 0.6–0.7; the expression of the enzyme was induced during 24 h at 16 °C with 0.2 mM isopropyl β-D-1-thiogalactopyranoside. The cells were harvested by centrifugation (5000× *g*, 20 min) and then resuspended in a buffer (20 mM HEPES-KOH (pH of 7.8), 40 mM NaCl) with the addition of a mixture of EDTA-free protease inhibitors (Inhibitor cocktail, Complete, Mannheim, Germany) followed by cell lysis by means of a French press. All the purification procedures were carried out at 4 °C. The homogenate was centrifuged at 40,000× *g* for 45 min. The supernatant was then passed through a 0.45 µm filter (Labfil, ALWSCI, Zhejiang, China) and the NaCl concentration in the supernatant was brought to 500 mM, whereupon the supernatant was mixed with Ni-Sepharose HP (Cytiva, GE Healthcare Life Sciences, Marlborough, MA, USA). The elution of the protein-containing fraction was carried out with a buffer composed of 500 mM NaCl, 20 mM HEPES-NaOH (pH of 7.8), and 600 mM imidazole. Then, the NaCl concentration in the eluent was brought to 150 mM by dilution with 20 mM HEPES-NaOH (pH of 7.8). The resulting solution was loaded on a 1 mL HiTrap-SP HP^TM^ column (Cytiva GE Healthcare Life Sciences, United States). Bound protein was eluted with a linear 150→1200 mM gradient of NaCl. The homogeneity of the protein was verified by SDS-PAGE (Figure 12); the protein concentration was measured on the NanoPhotometer (Implen, Munich, Germany). Glycerol was added to the isolated fractions up to 50% (*v*/*v*). Enzyme stock solutions were stored at −70 °C.

### 4.4. CD Spectra

These spectra were recorded on a Jasco J-600 spectropolarimeter (Jacso, Tokyo, Japan) at 25 °C in quartz cells with a 1 cm light path length. The concentration of ABH2 in the device cell was 20 µM. The experiments were conducted in a buffer consisting of 50 mM Tris-HCl with a pH of 8.0. The spectra were acquired at a bandwidth of 1.0 nm and a resolution of 1.0 nm at a scan speed of 50 nm/min. The scans were accumulated and automatically averaged.

### 4.5. The EMSA

WT ABH2, or one of its mutant forms, was first diluted with EMSA buffer (50 mM Tris-HCl with a pH of 8.0, 50 mM KCl, 10 mM MgCl_2_, 0.5 mM αKG, and 2 mM sodium ascorbate). In parallel, 6-carboxyfluorescein (FAM)-5′-labeled oligodeoxyribonucleotides containing m^1^A or m^3^C as damage were heated with the respective complementary strand at 95 °C for 3 min, then cooled to 22 °C. The binding reactions were initiated by adding 5 μL of the DNA sample to 5 μL of the protein samples. Standard binding reactions contained 1.2 µM of FAM-labeled damaged DNA duplex with protein concentrations between 0.4 and 12.8 µM. The reaction mixtures were incubated for 10 min at 37 °C, then cooled for 5 min on ice. After the glycerol concentration in a sample was brought to 5%, the reaction mixtures were loaded on a nondenaturing 10% polyacrylamide gel (70:1 acrylamide:bis-acrylamide) in 0.5× TBE buffer (1× TBE buffer: 90 mM Tris, 90 mM boric acid, 2.4 mM EDTA), and electrophoresis was run for approximately 35 min at 200 V in a cool box (at ~4–8 °C). The gels were visualized using a Molecular Imager ^®^VersaDoc™ MP Imaging System (Bio-Rad, Hercules, CA, USA) and the bands were quantified by scanning densitometry in the Gel-Pro Analyzer software, v.4.0 (Media Cybernetics, Rockville, MD, USA). The fraction of bound DNA [F = DNA_bound_/(DNA_bound_ + DNA_unbound_)] was plotted against the protein concentration, and the binding data were fitted to the Hill equation (Equation (1)) with the OriginPro 8 software (OriginLab, Northampton, MA, USA). Titration series (nine points) were implemented in triplicate.

(1) $$F=F_{u}+\frac{F_{b}-F_{u}}{1+{\left(\frac{K_{d}}{E_{0}}\right)}^{h}}$$

where F is the fraction of bound DNA; F_u_ and F_b_ are the initial and final levels of F, respectively; h is the Hill coefficient; K_d_ is the effective dissociation constant of the complex.

### 4.6. Methylated DNA Repair Activity Assays

FAM-5′-labeled oligonucleotides were subjected to experiments on the separation of the cleavage products by PAGE. The repair activity assays were carried out in reaction buffer (50 mM Tris-HCl (pH of 8.0), 50 mM KCl, 10.0 mM MgCl_2_, 1.0 mM αKG, 2.0 mM sodium ascorbate, and 40 μM (NH_4_)_2_Fe(SO_4_)_2_·6H_2_O). Analyses of the active demethylation of the DNA substrates containing m^1^A or m^3^C as a lesion were performed at 37 °C in a 10 µL reaction mixture for 30 min. The substrate concentration was 1.0 µM and the concentration of the enzyme was 1.0 µM as well. The reaction was initiated by the addition of the enzyme. After 30 min, the reaction mixture was subjected to precipitation with a 2% solution of LiClO_4_ in acetone.

In the analysis of the kinetics of the active demethylation product accumulation, the reaction between m^1^A- or m^3^C-containing DNA substrates and the WT enzyme or mutant forms was stopped after a certain period ranging from 30 s to 1 h.

To visualize the product of DNA demethylation, each precipitated sample was treated with 5 U of restriction endonuclease BstMB I (SibEnzyme, Novosibirsk, Russia), which recognizes the GATC sequence and is sensitive to DNA methylation. The digestion of the ABH2 reaction product by BstMB I was carried out for 30 min at 37 °C. The reaction with BstMB I was quenched with 5 µL of a gel-loading dye containing 7 M urea and 50 mM EDTA; then, the samples were loaded onto 20% (*w*/*v*) polyacrylamide gel containing 7 M urea. PAGE was performed at 55 °C and a voltage of 200–300 V. The gels were visualized using the Molecular Imager VersaDoc™ MP Imaging System (Bio-Rad, Hercules, CA, USA), and the bands were quantified by scanning densitometry in Gel-Pro Analyzer v.4.0 (Media Cybernetics, Rockville, MD, USA).

The data were analyzed via the following equation, under the assumption that an observed catalytic rate constant is a ratio of the initial velocity, *V*_0_, to the equilibrium concentration of the precatalytic complex [E·S]:

(2) $$k_{obs}=2V_{0}{\left(E_{0}+S_{0}+K_{d}+\sqrt{{\left(E_{0}+S_{0}+K_{d}\right)}^{2}-4E_{0}S_{0}}\right)}^{-1}$$

where *V*_0_ is initial velocity estimated as the initial slope of the kinetic curve obtained under steady-state reaction conditions, E_0_ and S_0_ are the total concentrations of the enzyme and DNA, and *K_d_* is an equilibrium dissociation constant calculated in the EMSA assay by means of Equation (1).

### 4.7. MD Simulations

Initial simulation structures were derived from available crystal structures of ABH2 cross-linked with DNA containing modified nucleotides everted into the enzyme active site in the presence of cofactor αKG and the Mn^2+^ ion: PDB ID: 3RZJ for m^3^C- and m^5^C-containing models and PDB ID: 3BUC and 3RZK for m^1^A models [32,39]. Poorly resolved amino acid residues were filled in using Modeller [53], cross-linking modifications were removed, and DNA duplex sequences were adjusted to match the experimental ones.

Amino acid sidechain protonation states were assigned on the H++ server [54]. Simulation setup and simulations were performed using the GROMACS MD package [55]. The starting structures were solvated and neutralized in a dodecahedral PBC box by means of TIP3P model water and 50 mM KCl JC ions [56,57]. An AMBER 14SB forcefield with OL15 corrections was used to describe the protein and the substrate DNA [58,59,60,61]. The AMBER forcefield parameters for αKG, m^3^C, and m^1^A were generated by Antechamber, with RESP charges calculated by the REDD server [62,63,64,65,66]. Conversion to GROMACS-compatible formats was performed with the ACPYPE tool [67]. The active-site Fe^2+^ ion was modeled as a nonbonded cationic dummy model [68].

The cutoff of nonbonded interactions was set to 1.0 nm, and long-range electrostatic interactions were processed by the PME method [69,70]. Covalent bonds involving hydrogen atoms were constrained using the LINCS solver [71]. Steepest-descent energy minimization was followed by 1 ns NVT and NPT equilibrations with restrained positions of all solute heavy atoms, with the help of the Bussi thermostat and Parrinello–Rahman barostat [72,73]. Postequilibration unrestrained MD simulations were run for 100 ns in triplicate, with one set each for m^3^C and m^1^A, and were extended up to 500 ns. Trajectory processing was performed using the integrated GROMACS toolset. Images were generated in the open-source version of the PyMOL viewer.

## Figures and Tables

**Table 1:** The list of amino acid residues forming contacts with three types of damaged bases inside the active site of WT ABH2.

	m^1^A	m^3^C	εA
Val99	Wall of damaged-base pocket	Wall of damaged-base pocket	Wall of damaged-base pocket
Val101	DNA-intercalating residue	DNA-intercalating residue	DNA-intercalating residue
Phe102	DNA-intercalating residue	DNA-intercalating residue	DNA-intercalating residue
Arg110	Coordination of damaged base (4.38 Å)	Coordination of damaged base (3.9 Å)	Coordination of damaged base (4.52 Å)
Tyr122	Hydrogen bond with damaged base	Hydrogen bond with damaged base	No direct interaction with damaged base
Phe124	Stacking with damaged base	Stacking with damaged base	Stacking with damaged base
Ser125	Wall of damaged-base pocket	Wall of damaged-base pocket	Coordination of damaged base (4.25 Å)
Ile168	Wall of damaged-base pocket	Wall of damaged-base pocket	Wall of damaged-base pocket
His171	Stacking with damaged base	Stacking with damaged base	Stacking with damaged base
Asp173	Hydrogen bond with damaged base	Hydrogen bond with damaged base	No direct interaction with damaged base
Glu175	Hydrogen bond with damaged base	Hydrogen bond with damaged base	No direct interaction with damaged base

**Table 2:** A summary of kinetic parameters of DNA demethylation by ABH2 enzymes, as determined under steady-state conditions.

	*K_d_*, µM	*k_obs_*, s^−1^	
WT	2.45 ± 0.07	0.071 ± 0.007	m^1^A
Y122A	2.11 ± 0.08	0.013 ± 0.006	
I168A	2.7 ± 0.4	0.02 ± 0.01	
D173A	2.10 ± 0.09	0.010 ± 0.003	
WT	3.5 ± 0.1	0.045 ± 0.007	m^3^C
Y122A	2.49 ± 0.06	0.005 ± 0.001	
I168A	4.1 ± 0.1	0.008 ± 0.002	
D173A	3.09 ± 0.07	0.0045 ± 0.0009	

**Table 3:** DNA substrates used in this study.

Shorthand	Sequence
m^1^A	5′-FAM-AGTTCAATG-m^1^A-TCTTCAT-3′ 3′-TCAAGTTACTAGAAGTA-5′
m^3^C	5′-FAM-AGTTCAATGAT-m^3^C-TTCAT-3′ 3′-TCAAGTTACTAGAAGTA-5′
GATC	5′-FAM-AGTTCAATGATCTTCAT-3′ 3′-TCAAGTTACTAGAAGTA-5′

## Data Availability

Experimental data are available upon request to N.A.K. Tel. +7-383-363-5174, E-mail: nikita.kuznetsov@niboch.nsc.ru.
